# Does having a usual primary care provider reduce patient self-referrals in rural China’s rural multi-tiered medical system? A retrospective study in Qianjiang District, China

**DOI:** 10.1186/s12913-017-2673-6

**Published:** 2017-11-28

**Authors:** Da Feng, Donglan Zhang, Boyang Li, Yan Zhang, Ray Serrano, Danxiang Shi, Yuan Liu, Liang Zhang

**Affiliations:** 1 School of Medicine and Health Management, Tongji Medical College, Huazhong University of Science and Technology, No.13 of Hangkong Road, Qiaokou District, Wuhan, Hubei Province China; 2 Department of Health Policy and Management, College of Public Health, University of Georgia, Athens, GA USA; 3 Department of Health Policy and Management, Rollins School of Public Health, Emory University, Atlanta, GA USA; 4 Outpatient Office, Shanghai First Maternity and Infant Hospital, Shanghai, China

**Keywords:** Usual primary care provider, Self-referral, Multi-tiered medical system

## Abstract

**Introduction:**

Within China’s multi-tiered medical system, many patients seek care in higher-tiered hospitals without a referral by a primary-care provider. This trend, generally referred to as patient self-referral behavior, may reduce the efficiency of the health care system. This study seeks to test the hypothesis that having a usual primary care provider could reduce patients’ self-referral behavior.

**Methods:**

We obtained medical records of 832 patients who were hospitalized for common respiratory diseases from township hospitals in Qianjiang District of Chongqing City during 2012–2014. Logit regressions were performed to examine the association between having a township hospital as a usual provider and self-referring to a county hospital after being discharged from a township hospital, while controlling for patients’ gender, age, income, education, severity of disease, distance to the nearest county hospital and the general quality of the township hospitals in their community. A propensity score weighting approach was applied.

**Results:**

We found that having a usual primary care provider was associated with a lower likelihood of self-referral (odds ratio = 0.58, 95% confidence interval [CI] =0.41–0.82), and a 9% (95% CI: -14%, − 3%) reduction in the probability of patients’ self-referral behavior.

**Discussion/conclusion:**

The results suggest that establishing a long-term relationship between patients and primary care providers may enhance the patient-physician relationship and reduce patients’ tendency for unnecessary use of medical resources.

**Electronic supplementary material:**

The online version of this article (10.1186/s12913-017-2673-6) contains supplementary material, which is available to authorized users.

## Background

By the end of the 1970s, China had established a multi-tiered medical system in rural areas, within which township hospitals provided primary and preventive care, whereas county hospitals provided specialty care for patients with severe conditions [1, 2]. During this period while this two-tiered system worked effectively to support both tiers of hospitals in providing care, life expectancy and general health significantly improved among the rural Chinese population [3]. In 1979, China reformed its economic structure from a centralized planned economy to a more market-based economy. These changes affected the health care system by instilling a competition-oriented mechanism that favored a hospital-centric model [4]. Since then, the two-tiered system largely collapsed and township hospitals have lost substantial public funding and support. Township hospitals and county hospitals started to operate independently and often competed with one another. Most township hospitals were unable to compete with larger county hospitals, as patients perceived their quality as substandard compared to county hospitals [5, 6]. As a result, patients viewed their primary care providers in township hospitals with distrust and over utilized medical services provided by county hospitals [7, 8].

### Study context: The 2009 revitalization of multi-tiered medical system

In 2009, China launched the largest health care reform and reintroduced the multi-tiered medical system as a viable solution to reduce overutilization of health care services in county hospitals in order to contain health care costs [9]. Seven years later, the quality of primary health care providers in township hospitals have significantly improved in line with national standards for treating patients with minor conditions [10, 11]. Nevertheless, patients still regard primary health care providers as providing only a limited range and lower quality of healthcare services [12]. According to the Fifth China National Health Service Survey in 2013, 67.4% of patients opted for care provided by county hospitals in rural areas [13]. Some studies found that in China’s rural areas, many patients prefer township hospitals as their usual sources of care [14]. However, rural residents visit county hospitals without a physician referral after having been seen at a township hospital. The findings indicate an unnecessary use of medical resources which has resulted in rising health care spending [15].

The importance of having a usual primary care provider in accessing health services has been well-documented [16, 17]. A number of studies in developed countries have shown that having a usual primary care provider is associated with greater patient satisfaction with their providers, more utilization of preventive services, lower utilization of emergency services and shorter hospital stays [18, 19]. A usual primary care provider was also associated with improved access to health care and better quality of care [16, 20, 21]. However, the effect of having a usual primary care provider on self-referral behavior in a multi-tiered care system has not been well understood and is especially under-studied in developing countries. For example, previous studies in the United States have shown that patients who self-refer to emergency departments are also more likely to bypass their primary care providers [22–24]. In China, one study found that distrust in healthcare providers may deter people from seeking care, and some have argued, albeit with scant evidence, distrusting primary hospitals leads to less utilization of primary care services and overutilization of specialty care in higher-tiered hospitals. Yet to date, there are few studies on patients’ self-referral behavior [25]. Existing literature on healthcare referrals in China has largely focused on providers’ referral rather than on patients’ self-referral [26]. Moreover, the main focus was often the association of referral behavior with socio-demographic factors, income, health insurance and distance from health service providers rather than usual primary care providers [27, 28].

In this study, we have used medical records and patient surveys to examine the relationship between having a usual primary care provider and self-referral behavior. A conceptual model based on the Anderson and Adey Model and the Health Belief Model (HBM) was applied to understand the determinants of self-referral behavior within China’s specific context. Self-referral in this study was defined specifically as patients who self-referred to secondary hospitals (county hospitals) without a professional referral from a primary care provider (township hospitals), after being treated by a primary care provider within 30 days. This study was conducted three years after the 2009 health care reform in Southwestern China, where primary care providers play an important role in providing rudimentary health care for rural patients.

## Methods

### Conceptual framework

**Fig. 1:**
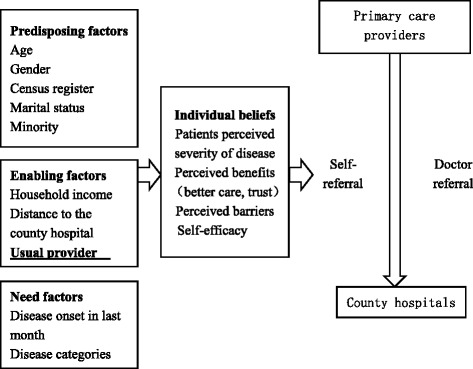
A Conceptual Framework for Assessing Patients’ Self-referral Behaviors within China’s two-tiered health system in rural region. We integrated the Anderson and Adey model with the health belief model to understand patients’ self-referral behavior. Both models have been widely used to analyze health services utilization. As shown in Fig. 1, individual belief is a mediator in the pathway between individual predisposing factors (i.e. age, gender, census registration status, and marital status), enabling factors (i.e. household income, and whether having a usual primary care provider), need factors (i.e. whether the same disease existed in the last month and whether the patient is in an acute condition) and patients’ health care seeking behavior

We integrated the Anderson and Adey model with the health belief model to understand patients’ self-referral behavior. Both models have been widely used to analyze health services utilization [22, 23, 29]. As shown in Fig. 1, individual beliefs include patients’ perceived severity of disease, their trust in primary care providers, perceived barriers and self-efficacy. First, whether patients trust their primary care providers may be influenced by the actual quality of care provided by the primary providers as well as by patients’ personal characteristics. Second, perceived barriers to obtain better and specialty care is one predictor of patient preference for higher-tiered hospitals. Third, patients may tend to proactively seek higher-tiered hospitals due to improved self-efficacy. Other factors that may have an impact on patients’ self-referral behavior but were overlooked in previous literature include distance to the nearest county hospital, and the distribution of multi-ethnic population. Furthermore, since all studied participants have been covered by the New Rural Cooperative Medical Scheme, we did not include insurance coverage as a necessary enabling factor. Finally, although not shown in this figure, factors that would influence whether patients have a usual primary care provider include age, gender, education, culture, family economic status, health status, perceived quality of health care and patients’ attitudes toward primary care providers [30, 31].

### Study design

To explain the relationship between having a usual primary care provider and patients’ self-referral behavior, we have collected cross-sectional data from medical records in Qianjiang district, Chongqing from Southwestern China in July 2015. To date, evidence on self-referral behavior has not been differentiated between unnecessary self-referral and appropriate self-referral for patients who have acute health conditions [32]. In this study, we have restricted the sample to inpatients who have been treated for respiratory diseases during 2012 to 2014, a common and prevalent disease which can be treated by primary health care providers based on their medical training. We have selected all inpatients with respiratory diseases from the New Rural Cooperative Medical System (NRCMS), and extracted their medical records from all township hospitals first. Then if they also sought admission services from county hospitals without professional recommendation after the firstly admitted in township hospital, their medical records were also extracted.

### Study settings and sampling

**Fig. 2:**
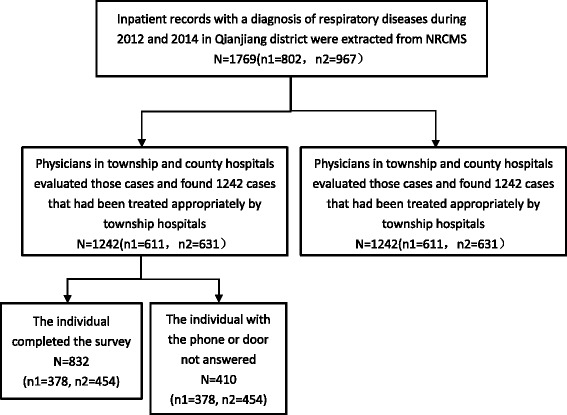
The sampling process of Survey Participants. We first extracted 1769 township inpatient medical records with a diagnosis of respiratory diseases during 2012 and 2014 in Qianjiang district. Physicians in township and county hospitals evaluated those cases and found 1242 cases that could be treated appropriately by township hospitals. We then conducted telephone surveys and household surveys among the 1242 cases to get patients’ individual, social and disease information which cannot be found in medical records. Finally, 832 cases were participated in these surveys, the response rate was 66.99%

Chongqing is a direct-controlled municipality located in the southwest region of China. Though named as a municipality, many Chongqing residents reside in rural areas. Moreover, Chongqing has a wide array of ethnic groups, representing the ethnic minorities in Southwestern China where most vulnerable population live. The sampling process was shown in Fig. 2. Notably, after using Chi-square test to explore differences of distribution on the general variables which occurred among three samples such as age, gender, no significant difference was observed. Out of the 832 cases, 378 (45.9%) were non self-referral patients and 454 (54.1%) were self-referral patients based on the one-on-one evaluation of the necessity of referring to county hospitals.

### Variables and measurement

The primary predictor of interest in this study was whether a patient reported having a usual primary care provider (township hospital). In this study, usual primary care provider was measured by asking the patients “in past 3 years, except for this visit, how many times did you seek health care in township hospitals and in county hospitals, respectively?” Those who visited primary hospitals more frequently than county hospitals were coded as 1 – “having a usual primary care provider”, and those who visited county hospitals more frequently or equally frequently or never visited a health care provider were coded as 0 – “not having a usual primary care provider”.

The dependent variable was the patient’s self-referral behavior, coded as 1 if they self-referred to a county hospital without a referral recommendation from a doctor after being hospitalized at a township hospital within 30 days, and coded as 0 if patients were admitted in a township hospital and did not visit a county hospital afterwards. We distinguished self-referral from doctor-referral because self-referral was considered unnecessary from the clinical standpoint, and could be mostly influenced by advices from friends and family members [33].

Confounding variables included gender, age (<45, 45–65, vs >65 years old), census registration status (rural vs urban), ethnicity (Han vs non-Han ethnic minority), disease onset in the last month (whether a patient had the same kind of disease in the last month), patient’s status at discharge (Improved, others such as stable and worse) disease category (Type A - ordinary acute diseases, Type B – acute disease with co-morbidities, Type C - common chronic diseases without comorbidities vs Type D - chronic diseases with comorbidities.) [34–38], and distance to a county hospital (<25, 25–50 vs >50 km) [39]. Since patients’ perceived quality of township hospitals may largely influences their choices, we further controlled a variable capacity of township hospitals, an index calculated using a comprehensive evaluation. The index included number of physicians and nurses, number of beds, hospital patient volume, outpatient volume, whether or not the providers are able to do the lower abdominal surgery in the township hospitals.

### Data collection

Trained medical students from Tongji Medical College, Huazhong University of Science and Technology collected the primary data in July 2015. Collected information included individual characteristics, social factors, and disease factors among patients with respiratory diseases who were discharged from primary-level hospitals (i.e., township hospitals), and some of them also self-referred to secondary-level hospitals (i.e., county hospitals) within 30 days. A list of patient names with individual information such as gender, age, were obtained from the NRCMS system, then linked with information such as census registration disease onset in last month, patient’s status at discharge, and disease category were extracted from their medical records stored in the township or county hospitals. Whether having a usual primary care provider or not, Household income per year and distance to county hospitals were collected by phone and household surveys. Due to few inpatients were hard to answer this question, we obtained this information from family members instead of the patients themselves. Respiratory diseases included in this study were acute bronchitis, chronic bronchitis, upper respiratory infection, tonsillitis (non-surgical) and ordinary pneumonia, which were illnesses that can be treated in primary hospitals according to the peer judgment from the physicians and the defined guideline for the scope of diagnosis and treatment in township hospitals.

The study was approved by the Ethics Committee of Tongji Medical College, Huazhong University of Science and Technology (IORG No: IORG0003571). Written consent forms were obtained from all participants prior to conducting interviews and participation in the study was entirely voluntary. Information collected for this study was securely stored and maintained with considerations of patient information confidentiality.

### Statistical analysis

Descriptive analysis was firstly performed to compare the differences in socio-demographic and other factors between the non-self-referral and self-referral patients. Chi-square tests and independent t-tests were used to test the statistical significance. Multi-variate logistic regression analysis was used to model the association between having a usual primary care provider and participants’ self-referral behavior, by specifying a different set of aforementioned covariates. Propensity-score weighting approach was applied to adjust for the observed difference in characteristics between those with a usual primary care provider and those without, therefore could better tease out the net influence of having a usual primary care provider on patients’ self-referral behavior. We selected patients’ age, sex, ethnicity, census registration status as the predisposing factors, household income, distance to county hospitals, usual providers as the enabling factors, and whether having the same disease in the last month, recovery condition in township hospitals as the need factors and capacity of township hospitals to calculate the propensity score. Based on the conceptual models in Fig. 1, we put the covariates into the model step by step. The usual primary care provider are the key factors we considered about, therefore, in the model 1, we only used usual provider to explain the relationship between usual primary care provider and whether or not self-referral. In model 2, we control the predisposing factors and need factors to see the net effect of the usual primary care provider on the people’s self-referral behaviors. In model 3, we control the need factors such as disease related the factors. Finally, we controlled the capacity of township hospitals to reflect the effects of usual primary care providers. Sample characteristics after Propensity Score Weighting shows that covariates are relatively balance once the weights are applied. (Additional file 1: Table S1) All reported *p*-values were two-sided, and statistical significance level (α) was set at 0.05. Stata 14.2 was used to perform the statistical analysis (StataCorp LP., College Station, TX).

## Results

### Characteristics of the self-referral patients

Table 1 presents the sample characteristics among the 832 patients by whether self-referred to a county hospital after being discharged from a township hospital. Self-referral patients were significantly and more likely to be male (*p* = 0.037), less than 65 years old (*p* < 0.001), Han population (*p* < 0.001), high-income (*p* < 0.001), not having a usual primary care provider (*p* < 0.001), having the same disease in the last month (<0.001), not very far from the nearest county hospital (*p* = 0.002), having acute conditions with comorbidities (*p* < 0.001), patient’s status at discharge with worse or stable (*p* < 0.001) and having a township hospital in the community with very poor/poor/fair capacity.

**Table 1:** Sample Characteristics among patients who were hospitalized with respiratory disease^i^

Characteristics	Participants	Non self-referrals^a^	Self-referrals^b^	*P* value^h^
	*n* = 832 (100%)	*n* = 378 (45.9%)	*n* = 454 (54.1%)	
Gender				0.037
Male	412(49.5)	172(45.5)	240(52.9)	
Female	420(50.5)	206(54.5)	214(47.1)	
Age				
< 45	256(30.8)	104(25.2)	152(36.2)	<0.001
45–65	316(37.9)	152(36.9)	164(39.0)	
> 65	260(31.3)	156(37.9)	104(24.8)	
Ethnicity				
Minorities^c^	412(49.5)	328(61.2)	84(28.4)	<0.001
Han population	420(50.5)	208(38.8)	212(71.6)	
Census registration				
Rural	654(78.6)	332(80.6)	322(76.7)	0.177
Urban	178(21.4)	80(19.4)	98(23.3)	
Household net income per year^d^				
Less than CNY15,000	322 (38.7)	176(42.7)	146(34.8)	<0.001
CNY15,000-CNY30,000	292(35.1)	156(37.9)	136(32.4)	
CNY30,000 and above	218(26.2)	80(19.4)	138(32.9)	
A usual primary care provider^e^				
Yes	496 (59.6)	276 (67.0)	220(52.4)	<0.001
No	336 (40.4)	136 (33.0)	200(47.6)	
Disease onset in last month				
Yes	368 (44.2)	112(27.2)	256(61.0)	<0.001
No	464 (55.8)	300(72.8)	164(39.0)	
Disease category^f^				
Type A - ordinary acute diseases	422(50.7)	236(57.3)	186(44.3)	<0.001
Type B - acute disease with co-morbidities	192(23.1)	40(9.7)	152(36.2)	
Type C - common chronic diseases without comorbidities	116(13.9)	80(19.4)	36(8.6)	
Type D - chronic diseases with comorbidities	102(12.3)	56(13.6)	46(11.0)	
Patient’s status at discharge				
Others	302(36.3)	106(25.7)	196(46.7)	<0.001
Improved	530(63.7)	306(74.3)	224(53.3)	
Distance to the county hospital (km)				0.002
< 25	264(31.7)	120(29.1)	144(34.3)	
25–50	394(47.4)	220(53.4)	174(41.4)	
> 50	174(20.9)	72(17.5)	102(24.3)	
Capacity of township hospitals^g^				
Very poor	202(24.3)	88(21.4)	114(27.1)	0.001
Poor	102(12.3)	48(11.6)	54(12.9)	
Fair	204(24.5)	88(21.4)	116(27.6)	
Good	324 (38.9)	188(45.6)	136(32.4)	

^a^Non self-referrals referred to patients who received health care from primary care providers - township hospitals

^b^Self-referrals were defined as patients who received care from township hospitals, and then sought care in higher-level hospitals (county hospitals) without referral from primary care physicians

^c^Minorities mainly include the Miao population and Tujia population

^d^Household net income per year is measured by net household income owned by the residents after subtracting related expenditures. 1 Chinese currency Yuan (CNY) = 0.15 U.S. Dollars

^e^Having a primary care provider as a usual provider was approximately measured as that in the past 3 years, patients visited primary care providers more than other hospitals; other providers or none was measured as patients visited higher level hospitals more often than primary care providers or had no particular preference for health care providers

^f^Disease category: Type A represents ordinary acute diseases; Type B refers to acute disease with co-morbidities; Type C refers to common chronic diseases without comorbidities; Type D is chronic diseases with comorbidities

^g^Patient’s status at discharge: Others included stable, worse

^h^Capacity of township hospitals: Four levels of capacity of township hospitals were measured via a comprehensive evaluation conducted by ××. The index included number of physicians and nurses, number of beds, hospital patient volume, outpatient volume, whether or not the providers are able to do the lower abdominal surgery in township hospitals

^i^Results shown as frequency (percentage). *P* values were calculated using Chi-square test

### Regression analysis results

Table 2 shows the results from several multi-variate logistic regressions, adjusting for different sets of confounding factors. Without adjusting for confounding variables, patients who had a usual primary care provider was associated with a lower likelihood of self-referral (Odds Ratio [OR] =0.54, 95% Confidence Interval [CI] =0.41, 0.72). While controlling for patients’ socio-demographic characteristics including age, sex, household income, ethnicity, census registration status and distance to county hospitals, the odds ratio of having a usual primary care provider on self-referral behavior was smaller but still statistically significant (OR = 0.60, 95% CI = 0.44, 0.82). Further adjusting for the large influence of disease onset in the last month, disease categories and patient’s status at discharge, having a usual primary care provider was still significantly associated with self-referral (OR = 0.53, 95% CI = 0.37,0.76). Finally, further adjusting for capacity of township hospitals did not change the main result (OR = 0.55, 95% CI = 0.39, 0.79).

**Table 2:** Regression analyses examining the association between having a usual provider and self-referral to higher-level hospitals among patients with respiratory disease^a^

Variables	Odds Ratio (95% Confidence Interval)			
	Model 1	Model 2	Model 3	Model 4
Usual provider^b^				
Primary care providers	0.54^***^ (0.41,0.72)	0.60^**^ (0.44,0.82)	0.53^***^ (0.37,0.76)	0.55^***^ (0.39,0.79)
Other providers or none	1.00	1.00	1.00	1.00
Age(years)				
> 65		0.44^***^ (0.28,0.68)	0.43^**^ (0.26,0.73)	0.46^**^ (0.27,0.77)
45–65		0.58^**^ (0.38,0.86)	0.68 (0.43,1.07)	0.73 (0.46,1.17)
< 45		1.00	1.00	1.00
Gender				.
Female		0.65^**^ (0.47,0.89)	0.72 (0.51,1.02)	0.73 (0.51,1.03)
Male		1.00	1.00	1.00
Household net income per year^c^				
CNY30,000 and above		3.85^***^ (2.51,5.92)	3.06^***^ (1.89,4.96)	2.89^***^ (1.78,4.70)
CNY15,000-CNY30,000		1.28 (0.88,1.86)	1.07 (0.71,1.62)	1.06 (0.70,1.60)
Less than CNY15,000		1.00	1.00	1.00
Ethnicity^d^				
Han population		4.44^***^ (3.16,6.23)	3.94^***^ (2.69,5.75)	3.971^***^ (2.71,5.82)
Minorities		1.00	1.00	1.00
Census registration				
Urban		1.35 (0.89,2.07)	2.04^**^ (1.26,3.31)	2.13^**^ (1.32,3.45)
Rural		1.00	1.00	1.00
Distance to county hospitals (km)				
> 50		1.184 (0.77,1.82)	0.84 (0.52,1.37)	0.73 (0.43,1.21)
25–50		0.688^*^ (0.48,0.98)	0.69 (0.46,1.03)	0.66^*^ (0.44,1.00)
< 25		1.00	1.00	1.00
Disease onset in last month				
Yes			4.334^***^ (3.00,6.26)	4.247^***^ (2.92,6.18)
No			1.00	1.00
Disease categories^e^				
Type B			4.16^***^ (2.62,6.60)	4.43^***^(2.76,7.09)
Type C			0.85 (0.51,1.43)	0.86[0.51,1.46]
Type D			1.11 (0.66,1.89)	1.15 (0.67,1.96)
Type A			1.00	1.00
Patient’s status at discharge^f^				
Others			1.66^**^ (1.14,2.42)	1.59^*^ (1.08,2.33)
Improved			1.00	1.00
Capacity of township hospitals^g^				
Very poor				1.40 (0.88,2.24)
Poor				1.58 (0.90,2.80)
Fair				1.24 (0.79,1.96)
Good				1.00
N	832	832	832	832

^a^Non self-referrals referred to patients who received health care from primary care providers - township hospitals; Self-referrals were defined as patients who received care from township hospitals, and then sought care in higher-level hospitals (county hospitals) without referral from primary care physicians

^b^Having primary care providers as usual provider was approximately measured as that in the past 3 years, patients visited primary care providers more than other hospitals; other providers or none was measured as patients visited higher level hospitals more often than primary care providers or had no particular preference for health care providers

^c^Household net income per year is measured by net household income owned by the residents after subtracting related expenditures. 1 Chinese currency Yuan (CNY) = 0.15 U.S Dollars

^d^Minorities mainly include the Miao population and Tujia population

^e^Disease category: Type A represents ordinary acute diseases; Type B refers to acute disease with co-morbidities; Type C refers to common chronic diseases without comorbidities; Type D is chronic diseases with comorbidities

^f^Patient’s status at discharge: Others included stable, worse

^g^Capacity of township hospitals: Four levels of capacity of township hospitals were measured via a comprehensive evaluation conducted by ××. The index included number of physicians and nurses, number of beds, hospital patient volume, outpatient volume, whether or not the providers are able to do the lower abdominal surgery in township hospitals

* *p* < 0.05, ** *p* < 0.01, *** *p* < 0.001

Table 3 presents the results from the propensity-score weighted regression, adjusting for all relevant covariates. As this model produced the most consistent estimate, we calculated both odds ratios and marginal effects. Results from the propensity-score weighted regression showed that having a usual primary care provider was significantly associated with a lower odds of self-referral to county hospitals (OR = 0.58, 95% CI = 0.41, 0.82), and the corresponding marginal effect was −0.09 (95% CI = −0.14, −0.03), indicating that having a usual primary care provider reduced the likelihood of self-referral by 9 percentage points. With regards to the covariates, we found that patients aged 45 and older, females and living far away from county hospitals were less likely to self-referral. But Han population, urban population, those with the same disease in the last month, having acute or chronic condition with comorbidities, and those having a township hospital with fair capacity were more likely to self-referral to visit a county hospital after being treated at a township hospital.

**Table 3:** Propensity-score weighting regression examining the association between having a usual provider and self-referral to higher-level hospitals among patients with respiratory disease^a^

Variables	Odds Ratio (95% Confidence Interval)	Marginal Effect^b^ (95% Confidence Interval)
Usual provider^c^		
Primary care providers	0.58** (0.41, 0.82)	−0.09** (−0.14, −0.03)
Other providers or none	1.00	
Age (years)		
> 65	0.70 (0.42,1.19)	−0.17** (−0.26, −0.06)
45–65	0.37*** (0.20,0.70)	−0.06 (−0.14, 0.03)
< 45	1.00	
Gender		
Female	0.63* (0.43,0.91)	−0.07* (−0.13, −0.02)
Male	1.00	
Household net income per year^d^		
CNY30,000 and above	3.52*** (2.03,6.12)	0.21*** (0.12, 0.29)
CNY15,000-CNY30,000	0.99 (0.65,1.50)	−0.00 (−0.07, 0.07)
Less than CNY15,000	1.00	
Ethnicity^e^		
Han population	4.47*** (3.13,6.40)	0.25*** (0.20, 0.31)
Minorities	1.00	
Census registration		
Urban	2.43** (1.45,4.07)	0.14** (0.06, 0.22)
Rural	1.00	
Distance to county hospitals (km)		
> = 50	0.62 (0.41,0.95)	−0.08** (−0.14, −0.01)
25–50	0.65** (0.38,1.13)	−0.07 (−0.15, 0.02)
< 25	1.00	
Disease onset in last month		
Yes	3.95*** (2.59,6.02)	0.23*** (0.17, 0.30)
No	1.00	
Disease categories^f^		
Type B	5.61*** (3.36,9.37)	0.29*** (0.22, 0.37)
Type C	0.78 (0.43,1.38)	−0.04 (−0.14, 0.06)
Type D	1.94* (1.12,3.35)	0.12* (0.02, 0.21)
Type A	1.00	
Patient’s status at discharge^g^		
Others	1.46(0.98,2.17)	0.06(−0.01,0.13)
Improved	1.00	
Capacity of township hospitals^h^		
Very poor	1.35 (0.85,2.16)	0.10 (−0.01, 0.18)
Poor	1.66 (0.94,2.93)	0.08 (−0.01, 0.17)
Fair	1.82* (1.10,3.00)	0.03* (0.01, 0.12)
Good	1.00	
N	832	832

^a^Non self-referrals referred to patients who received health care from primary care providers - township hospitals

^b^Self-referrals were defined as patients who received care from township hospitals, and then sought care in higher-level hospitals (county hospitals) without referral from primary care physicians

^c^Minorities mainly include the Miao population and Tujia population

^d^Household net income per year is measured by net household income owned by the residents after subtracting related expenditures. 1 Chinese currency Yuan (CNY) = 0.15 U.S. Dollars

^e^Having a primary care provider as a usual provider was approximately measured as that in the past 3 years, patients visited primary care providers more than other hospitals; other providers or none was measured as patients visited higher level hospitals more often than primary care providers or had no particular preference for health care providers

^f^Disease category: Type A represents ordinary acute diseases; Type B refers to acute disease with co-morbidities; Type C refers to common chronic diseases without comorbidities; Type D is chronic diseases with comorbidities

^g^Patient’s status at discharge: Others included stable, worse

^h^Capacity of township hospitals: Four levels of capacity of township hospitals were measured via a comprehensive evaluation conducted by ××. The index included number of physicians and nurses, number of beds, hospital patient volume, outpatient volume, whether or not the providers are able to do the lower abdominal surgery in township hospitals

* *P* < 0.05; ** *P* < 0.01; *** *P* < 0.001

## Discussion

This study provides evidence on self-referral behavior and its association with having a usual primary care provider three years after Chinese government re-introduced a multi-tiered medical system in its recent health care overhaul. We identified that patients who reported having a township hospital as their usual provider were significantly less likely to self-refer to a county hospital, and thus over utilized medical resources.

The finding, albeit observed in a relatively small sample from a rural region in the Southwestern China, was consistent when we specified different sets of covariates, and was bolstered with a cautious adjustment of relevant confounders and the use of a propensity score weighting approach. Having a usual primary care provider indicated that patients enjoyed a high level of continuity of care with a regular primary care physician. Patients who had established a close relationship with primary care providers may be more willing to seek preventive care at the township hospitals [14, 40, 41]. In addition, a close physician-patient relationship could be explained by the fact that providers at township hospitals were more familiar with the patients and their medical histories, thus reducing patients’ incentives to find another doctor at a higher-tiered hospital to confirm the diagnosis and treatment [8, 24].

Studies in other countries have shown the impact of having a general practitioner in reducing self-referral behavior in a similar hierarchical care system or a managed care system. For example, in the United States, some insurance plans such as Health Maintenance Organizations (HMO) require patients to see a primary care physician (PCP) and get a referral from their PCPs to visit a specialist [42]. But patients were also allowed or given the choice to self-refer to see a specialist in certain circumstances. A study on American Point-of-Service Health Plans reported that patients who self-referred to a specialist were less likely to have continuity of care with their PCPs [33]. And those patients tended to access specialty care directly or reported relationship problems with their PCPs. In many European countries that have a General Practitioners (GP) system, patient self-referral is less a challenge, and usually GPs, also named gatekeepers, are the ones who make professional judgement and refer patients to a specialist if necessary [43, 44]. In the real situation of China’s integrated medical system, China has scanned the policy “Contracted Family Doctors” of Community Health Center. Community health center doctors as a kind of usual primary care providers, it could further promote people using more primary hospital service which could significant contribute to reduce the self-referral behaviors. Majority of the researches in those countries focused on physicians’ referral pattern and referral network and how it may affect patient outcomes [45]. Whereas in the Chinese context, self-referral is much more prevalent, even though a multi-tier medical system had been established decades ago.

Self-referral behavior may reveal that patients have a lack of trust in the quality of care provided by the township hospitals, and may be unaware of the policy change that has resulted in substantial improvement in the capacity of township hospitals in treating non-critical conditions. A previous study actually showed that patient self-referral choice did not correlate with higher quality of care. To the opposite, self-referral may lead to lower quality of health care, higher medical cost and treatment delay [32]. Having a township hospital as a usual primary care provider could potentially change patients’ beliefs about the care provided by township hospitals, according to the conceptual model, and improve their perceived benefits while getting treatment from township hospitals.

With respect to individual characteristics, patients who were male, younger, Han ethnicity, with urban registered permanent residence, and having higher household income were more likely to self-refer to a higher-tiered hospital. This may underscore the importance of trust between patients, especially those with a relatively high socio-economic status, and primary care providers in township hospitals. The perception that the township hospitals were of inferior quality compared to higher-tiered hospitals may not be easily changed and may have a long-term effect on patients’ healthcare seeking behavior. More education efforts from the public sectors are needed to change this “status quo” bias whereby patients stick to their previous preferences. The results also showed that although patients who had a more acute condition tended to self-refer, those who had the same disease condition in the last month were also more likely to self-refer. It is likely that disease recurrence in a month largely influenced patients’ perceptions that their conditions were not well-treated in township hospitals, and increased their tendency to seek more effective treatment at higher-tiered hospitals [46].

There were several notable limitations in this study. First, despite medical records and hospital-based administrative information were obtained, measurement errors may occur when we collected information from various sources. In particular, the surrogate measure on usual primary care providers was reported by patients and their family members, thus recall bias may have taken effect. Nevertheless, we cross-checked most information with the health care providers and ascertained the self-referral measure from the NRCMS electronic system, which largely improved its accuracy. Second, the study focused only on patients who were hospitalized with common respiratory illnesses to reduce the threat to internal validity, whereby different disease condition may cause the associations to differ. It, however, also reduced the generalizability of our findings to patients who were treated for other diseases. In addition, our sample was confined to those living in the Qianjiang District in Southwestern China, which cannot be generalized to other places in the country. Finally, the study did not compare the health and cost outcomes between self-referral patients to non-self-referral patients. Future studies should examine how self-referral behavior affects health outcomes and medical costs, and highlight the necessity or benefits to reduce self-referral behavior.

## Conclusions

Township hospitals in China’s rural areas remained a critical, yet largely underutilized component of the country’s multi-tiered medical system. This study suggests that establishing a long-term relationship with a township hospital may reduce patient self-referral behavior, and may strengthen the role of township hospitals to provide the essential and basic health services to the vast rural population. Insurance design should incentivize rural patients to use primary care services in township hospitals and thus establish a close relationship with providers at township hospitals, particularly in their residential region. Funding and strategies from local public sectors are needed to further strengthen the quality and capacity of primary care providers. Educational efforts using billboards and mass media might be good choice to change patients’ biased perception towards township hospitals [47–49].

## Additional file

Additional file 1: Table S1. Sample Characteristics after Propensity Score Weighting disease. (DOCX 17 kb)

## Abbreviations

CIs: 95% confidence intervals
NRCMs: New Rural Cooperative Medical System
OR: Adjusted odds ratio
