# Supplementary material for: Does having a usual primary care provider reduce patient self-referrals in rural China’s rural multi-tiered medical system? A retrospective study in Qianjiang District, China
Source: BMC Health Serv Res. 2017 Nov 28;17:778. doi: 10.1186/s12913-017-2673-6 (PMC5704594; doi:10.1186/s12913-017-2673-6)
Supplement: Additional file 1: Table S1. — Sample Characteristics after Propensity Score Weighting disease. (DOCX 17 kb) [file 12913_2017_2673_MOESM1_ESM.docx]

**Dear reviewers,**

Thank you for your valuable comments and suggestions to our work. Please find our responses to your comments below (highlighted in green). Revisions have been made accordingly in our manuscript. And the reference has been revise based on the QC results’ report.

1. Please consider the list of authors as it currently stands with reference to our guidelines regarding qualification for authorship (<http://www.biomedcentral.com/submissions/editorial-policies#authorship).>

Currently, the contributions of authors BL, YZ, DS, RS, YL are not clear, and do not automatically qualify them for authorship. Please provide clarification on their contributions, or remove their names from the list of authors and place them in the “Acknowledgements” section instead. Please note that any changes in the list of authors requires the completion of the “Change in authorship” form.

Acquisition of funding, collection of data, writing of the manuscript or general supervision of the research group - alone - does not usually justify authorship.

Thanks for your comments. We have revise the contributions of authors and provide clarification on their contributions from line 8 to 15 in page 11.

2. Please remove the Figures and Appendix Table from the manuscript file, and upload these as separate files.

Thanks for your comments. We have remove the figures and appendix table from the manuscript.

3. Please upload the Appendix Table as a separate supplementary file.

Thanks for your comments. We have upload the appendix table as a separate supplementary.

4. Please add a section "Additional files" (after the References/Figure legends) where you list the following information for each additional/supplementary file in the file inventory:

- File name (e.g. Additional file 1)

- Title of data

- Description of data

Thanks for your comments. We have add the “additional files after references in page 14.

5. Figures should be provided as separate files, and each figure of a manuscript should be submitted as a single file.

Please ensure that all figures/tables and supplementary files are cited within the text. Any items which are not cited may be deleted by our production department upon publication.

Thanks for your comments. We have refer all figures/tables and supplementary files cited within the text.

6. Please provide figure titles/legends under a separate heading of 'Figure Legends' after the References. If Figure titles/legends are within the main text of the manuscript, please move them.

Figure files should contain only the image/graphic, as well as any associated keys/annotations. If titles/legends are present within the figure files, please remove them.

Thanks for your comments. We have remove the figure titles/legends out of the main text in the manuscript.

7. At this stage, please upload your manuscript as a single, final, clean version that does not contain any tracked changes, comments, highlights, strikethroughs or text in different colours. All relevant tables/figures/additional files should also be clean versions. Figures (and additional files) should remain uploaded as separate files.

Thanks for your comments. We have make the change.
